# Botulinum Toxin Induced Atrophy: An Uncharted Territory

**DOI:** 10.3390/toxins10080313

**Published:** 2018-08-02

**Authors:** Mehri Salari, Soumya Sharma, Mandar S. Jog

**Affiliations:** 1 Department of Clinical Neurological Sciences, London Health Sciences Centre, Western University, 339 Windermere Road, A10-026, London, ON N6A 5A5, Canada; mehri.salari@gmail.com (M.S.); soumya0325@gmail.com (S.S.); 2 Functional Neurosurgery Research Center, Shohada Tajrish Neurosurgical Center of Excellence, Shahid Beheshti University of Medical Sciences, 19839-63113 Tehran, Iran

**Keywords:** botulinum toxin, atrophy, SNARE

## Abstract

Botulinum neurotoxins (BoNTs) produce local chemo-denervation by cleaving soluble N-ethylmaleimide-sensitive factor activating protein receptor (SNARE) proteins. Botulinum neurotoxins are therapeutically indicated in several neurological disorders and have been in use for three decades. The long-term efficacy, safety, and side effects of BoNTs have been well documented in the literature. However, the development of muscle atrophy following chronic exposure to BoNTs has not received sufficient attention. Muscle atrophy is not only cosmetically distressing, but also has an impact on future injections. An extensive literature search was conducted on atrophy and mechanisms of atrophy. Five hundred and four relevant articles in the English language were reviewed. This review revealed the surprising lack of documentation of atrophy within the literature. In addition, as demonstrated in this review, the mechanisms and the clinical factors that may lead to atrophy have also been poorly studied. However, even with this limited information it is possible to indicate factors that could modify the clinical approach to botulinum toxin injections. This review highlights the need for further study of atrophy following BoNT injections.

## 1. Introduction

Botulinum neurotoxins (BoNTs) have been injected for medical and cosmetic indications since 1977 [1]. As a result of local chemo-denervation, BoNTs impair neuromuscular transmission thereby reducing muscle contractility. This phenomenon translates into BoNTs being an effective treatment for a variety of movement disorders and other neurological and non-neurological conditions. Several studies have indicated its effectiveness in the treatment of disorders such as dystonia, post stroke spasticity, and cerebral palsy to name a few [2,3,4,5,6,7,8,9]. The side effect profile of long-term botulinum toxin injections has been well documented, especially in individuals with dystonia and spasticity [2,3,4,5,6,7,8,9]. A retrospective longitudinal descriptive analysis by Ramirez-Castenada et al. of 89 subjects with dystonia showed that the most common side effects were dysphagia, ptosis, and neck weakness which has been duplicated in several other studies [3,10,11,12]. However, the literature on BoNT-induced muscle atrophy as an adverse reaction of chronic, repeated injections is sparse, as this side effect was not documented. Some animal studies have focused on the atrophy-inducing effects of BoNTs; however, only a few case reports or case series in humans are available describing muscle atrophy as either a wanted or untoward effect [13,14,15,16,17,18,19,20,21,22]. A recent review by Durand and colleagues [23] addressed the issue of BoNT-induced muscle atrophy in human subjects, but they were unable to formulate any conclusions about the mechanisms of BoNT-induced muscle atrophy. Understanding muscle atrophy and the underlying mechanisms could directly influence the clinical judgment of muscle targeting and dosing for the injector. In order to address this important topic, this review focuses on several issues regarding BoNT-induced atrophy. The absence of documentation of muscle atrophy in the clinical literature is made obvious followed by a discussion of the mechanisms of atrophy after BoNT injections. A comprehensive overview of factors including toxin characteristics, neuromuscular junction (NMJ), and muscle properties, which contribute to muscle atrophy is presented, and suggestions for how these factors can be used by clinicians to modify their BoNT injections are provided.

## 2. Method

Electronic search of English language literature was conducted, and articles published up to June 2018 were included. The following databases were searched: Pubmed/MEDLINE, Embase, Cochrane, Proquest, Web of science, Ovid, and Scopus. The key words searched were as follows: muscle atrophy, muscle structural proteins, muscle fat deposition, mitochondrial dysfunction, muscle denervation, axonal re-innervation, muscle fiber type, muscle fiber composition, satellite cell, muscle spindle, blood supply, blood vessel; plus, botulinum toxin. The search produced 504 articles excluding the non-English articles and articles without full-text, and all these papers were reviewed. Papers that had sufficient details to draw conclusions regarding the mechanism of muscle atrophy were referenced.

## 3. Influence of Toxin Serotype on Muscle Atrophy

Botulinum neurotoxins are produced by bacteria belonging to the genus *Clostridium* and there are seven recognised serotypes (BoNT/A to /G) with the recent addition of another serotype, BoNT/X [24,25,26,27]. BoNTs are composed of a light chain (LC, ~50 kDa) and a heavy chain (HC, ~100 kDa) connected via a disulfide bond. BoNTs exert their effect by binding to the pre-synaptic neuronal membrane followed by internalization and cleavage of different synaptic vesicle protein termed soluble N-ethylmaleimide-sensitive factor activating protein receptor (SNARE) proteins [25]. However, irrespective of which synaptic vesicle protein is cleaved, the fusion of synaptic vesicles to plasma membrane is interrupted thereby preventing neurotransmitter release at the neuro muscular junction [24]. Several BoNTs are commercially available for therapeutic use and include BoNT/A, BoNT/B, and BoNT/C [28]. Of these, the most commonly used are BoNT/A and BoNT/B [29,30].

The structural differences between the various serotypes influence which SNARE protein is cleaved, as well as the site of cleavage of the SNARE protein, and this could potentially impact their propensity to produce atrophy. The light chain of BoNT/A cleaves SNAP-25 (Synaptosomal associated protein-25) while BoNT/B cleaves synaptobrevin, which is a vesicle protein also known as VAMP (vesicle associated membrane protein). The site of cleavage of SNAP-25 by BoNT/A is such that it removes nine amino acids forming a 197-amino acid fragment (P197), which then competes with the un-cleaved SNAP-25 at the nerve terminal thereby prolonging the neuromuscular junction (NMJ) block [31]. This could explain the longer duration of action of BoNT/A, leading to a sustained period of muscle denervation resulting in higher likelihood of muscle atrophy. In fact, two studies have indicated that the duration of muscle paralysis with BoNT/B is shorter when compared to BoNT/A [29,32]. Also, a single study by Amjad and colleagues [33] on ten subjects with spasticity has shown that BoNT/A causes a greater rate of muscle atrophy when compared with BoNT/B.

The influence of toxin serotype on the development of atrophy could also depend upon the anatomical expression of the SNARE proteins. SNAP-25 is expressed not only at the NMJ, but also in the axonal growth cones and in the brain where it plays an important function in axonal growth. Therefore, in addition to cleavage of SNAP-25 in the presynaptic nerve terminal, BoNT/A also cleaves this SNARE protein in the axonal growth cones as well as centrally in the brain by the mechanism of retrograde transport. This in turn inhibits axonal growth both centrally and peripherally, thus interfering with muscle re-innervation leading to atrophy following BoNT/A injections [34,35,36,37,38]. On the contrary, BoNT/A has been shown to have neuritogenic properties, that is, it is capable of inducing neurite sprouting which cannot be purely attributed to its mechanism of action of chemo-denervation [39,40,41]. Wang et al. [39] demonstrated that following BoNT/A injections intrathecally in rats with spinal cord injuries, there was upregulation of expression of neurite growth factors such as growth associated protein 43 (p-GAP 43) and superior cervical ganglion 10 (SCG 10). The interplay of these two differing influences of BoNT/A on neurite regeneration would affect the development of atrophy with this serotype which needs to be further explored.

## 4. Factors Influencing Muscle Re-Innervation after BoNT Induced Paralysis

Sprouting is the process by which the functional recovery of neuromuscular transmission occurs following chemodenervation by BoNTs. Interruption of sprouting could leave the muscles “functionally denervated” leading to atrophy [42,43,44,45]. Several elements influence sprouting, such as Insulin like Growth Factor (IGF)-1, Schwann cells, motor axon length and fiber type composition.

Muscles paralyzed by BoNTs up regulate the expression of IGF-1, which plays a significant role in induction of sprouting and elongation of regenerating axons [42,46,47]. The expression of IGF-1 in the muscles has been found to be regulated by estrogen [47]. A study demonstrated that the gene expression of IGF-1 in skeletal muscle was higher in premenopausal women compared to postmenopausal women. However, when the comparison was made between post-menopausal monozygotic twins discordant for hormone replacement therapy (HRT), those women not receiving HRT had lower expression of IGF-1 gene [48]. Hence, the hormonal status of a woman could potentially influence sprouting following BoNT-induced paralysis, thus impacting re-innervation of the muscle.

Schwann cells initiate and guide the growth of axon sprouts that leads to muscle re-innervation [49]. The suggestion that BoNT/A enhances Schwann cells proliferation comes from an experiment conducted by Marinelli and colleagues [50]. They demonstrated that Schwann cells, when cultured in the presence of BoNT/A showed a significantly decreased release of acetylcholine (ACh) which implied that BoNT/A, had the capacity to block the release of ACh. This reduced levels of Ach, which in turn increases the level of Neuregulin-1, a factor needed for Schwann cell proliferation and maturation [50]. Therefore, BoNT/A may have a positive effect on Schwann cell proliferation and factors that decrease the number of available Schwann cells at the NMJ, for instance advancing age may influence effective sprouting [51].

The amount of sprouting also depends on the fiber type composition of the muscles. Skeletal muscles are composed of three types of fibers including type I, IIa, and IIb, which are also known as slow fiber, fast fatigable, and fast fatigue resistant fiber, respectively [52]. Type I fibers tend to develop early, multiple and robust re-innervation, compared to type IIb fibers after BoNT-induced paralysis [53,54,55]. Hence, it could be postulated that muscles abundant in type I fibers recover better due to early and more extensive sprouting.

Another determinant for sprouting could be the motor axon length. Motor axons innervating proximal muscles are known to sprout more robustly compared to those innervating distal muscles [56].

Therefore, the process of re-innervation after BoNT injection seems to be influenced by several factors, and these need to be taken into consideration while injecting an individual with BoNT. Thus, for instance, certain modifiable parameters such as toxin dose, toxin type, and frequency of injections could be adjusted when injecting distal muscles or those muscles that have predominantly type IIb fibers in order to lessen atrophy in susceptible patients. Currently, these nuances are not considered by injectors.

## 5. Skeletal Muscle Fiber Type Composition and Its Influence on BoNT-Induced Atrophy

As already elaborated above, skeletal muscles are composed of three types of fibers: type I, IIa, and IIb. The fiber type composition as well as fiber size of different muscles varies depending upon its function. Therefore, injections of BoNTs may produce differential atrophy due to this discrepancy in fiber type composition. The proportion of fiber types may also vary between individuals as those who express a higher proportion of type I fibers in one muscle are likely to express the same in other muscles [57]. Muscles such as vastus lateralis and sternocleidomastoid (SCM) have a lower proportion of type I fibers and a higher proportion of type IIb fibers compared to splenius capitis, trapezius, and scalenus medius. A study on New Zealand White rabbits by Fortuna et al. [14] showed that following BoNT injections into quadriceps femoris, there was a reduction in the muscle mass which, interestingly, varied between the different muscles that constitute quadriceps femoris. Vastus lateralis, which has a higher proportion of type IIb fibers, had more atrophy than vastus medialis, which has a comparatively lower amount of type IIb fibers [14]. Not only the fiber type composition, but also the size of the fibers differs in different muscle groups. For example, all fiber types in the vastus lateralis have a larger cross-sectional area compared to neck muscles such as splenius capitis and scalenus medius [54].

Several factors seem to influence the balance between the type I and type IIb fiber expression in skeletal muscles; these include muscle loading conditions, age, gender, race, and body weight composition. A study by Raoul et al. [58] found that in individuals with asymmetrical mandible morphology (lateral deviation), the masseter muscle phenotype composition was different between the two sides with proportion of type II fibers higher on the side of deviation. Therefore, in the same individual with an asymmetry in the mandible structure, BoNT could produce a differential effect on the masseter muscle due to variance in muscle loading conditions. Another important consideration is age, as type IIb fibers undergo denervation and atrophy as well as decrease in the cross-sectional area with advancing age [54,55,59]. Androgen receptors (ARs), which are expressed in skeletal muscles in males, could have an important role in determining the skeletal muscle fiber type, biasing towards type I fibers [60]. On the other hand, obesity could decrease the levels of type I fibers in skeletal muscles [61]. Race also influences the fiber type distribution in obese individuals. Obese African-American women have a lower percentage of type I fibers and higher type IIb than obese Caucasian women [62]. Since type I fibers have a better tendency to regenerate following chemo-denervation, the body fat composition as well as race and gender could potentially determine the level of atrophy following toxin injections. These factors of differing race/gender/age and muscle composition should be considered for further optimization of BoNT injection effects.

## 6. Satellite Cells and Their Role in Toxin-Induced Atrophy

Satellite cells (SCs) are stem cells in adult muscles and help in regeneration of the muscles. Several factors including the location of the muscles (cranial or appendicular) in which they reside, and their embryonic origin determine the capacity of these SCs to proliferate and differentiate after BoNT injection [63,64]. The SCs of the extra-ocular muscles (EOMs) are unique, since unlike limb muscles they continuously proliferate and regenerate irrespective of any external stimuli such as injury [64,65]. On the other hand, masseters, which are also cranial muscles, have poor regenerative capacity after an external injury compared to limb musculature [66]. The difference in the regenerative capacity of these two cranial muscles can be explained by the origin of the SCs. The SCs in masseter but not in the EOMs are derived from Is11 cell lineages [66,67]. Is11 is a negative regulator of myogenic differentiation and inhibits expression of muscle differentiation markers, myogenin, and myosin heavy chain. Also, self-renewal capacity of SCs is low in masseters which negatively impacts myogenic proliferation after BoNT injection [68].

Hence injectors may need to be aware of this differential SCs proliferation capacity when selecting a muscle which is cranially or spinally innervated and adjust the dose, type, and interval of BoNT injection accordingly.

## 7. Mitochondrial Dysfunction Following Botulinum Toxin Injection

Mitochondria are implicated in the production of reactive oxygen species (ROS) in inactive muscles. This elevated production of ROS can lead to muscle atrophy by the process of proteolysis and suppression of protein synthesis [69]. Botulinum neurotoxin injections have been shown to alter the mitochondrial structure and to downregulate the genes for ROS scavenger in skeletal muscles [70,71]. These alterations lead to increased oxidative stress in the muscles injected with BoNT.

Oxidative stress is known to impair synaptic transmission by targeting the SNARE complex, particularly SNAP-25 [72]. Thus, SNAP-25 in the newly formed sprouts could undergo spontaneous oxidation thereby interfering with muscle re-innervation leading to atrophy [72].

It is possible that some individuals might be more susceptible to the effect of toxins due to pre-existing underlying mitochondrial dysfunction leading to more severe atrophy. Patients with sub-clinical mitochondrial cytopathy had more severe weakness after BoNT injection [73]. More commonly, diseases such as type 2 diabetes and obesity [74] that affect mitochondrial morphology and number could possibly increase the risk of atrophy after BoNT injection. Currently, these variables are not considered while determining BoNT dosing.

## 8. Muscle Spindles and Their Role in BoNT-Induced Atrophy

The muscle spindle is a sensory organ enclosed in a capsule and is composed of striated muscle fibers (intrafusal fibers). These organs receive afferent (Ia and II) and efferent nerve endings (gamma motor neuron) [75]. The location, number, and the depth of spindles differs between muscle groups. Neck muscles have a higher number of spindles compared to shoulder girdle [76]. Also, amongst the neck muscles, the intermediate group (longissimus capitis and cervicis muscles) have a higher number of spindles when compared to the medial group (semispinaslis, spinalis, and multifidus muscles), and the lateral group (lateral column iliocostocervicalis) [77].

The location of the spindles differs between different group of muscles. In the sub-occipital muscles, these spindles are situated superficially, whereas spindles in the postvertebral muscles of the thoracic, lumbar, and sacral regions are located deeper [77]. Furthermore, proximal muscles (arms) have more spindles compared to distal muscles (hand), however this difference is not noted in the lower limbs [76].

Botulinum neurotoxins have a direct influence on these spindle organs; they can produce atrophy in intrafusal fibers similar to that found in extrafusal fibers, and electrophysiological recordings also show that the afferent discharges are reduced from the intrafusal fibers after BoNT injections. The efferent nerve endings (gamma motor neurons) could be blocked by BoNT, thereby decreasing the afferent signal from the muscle spindle, subsequently reducing the muscle tone by reflex inhibition without reducing the muscle strength [78].

To the best of our knowledge, no studies have compared the amount of atrophy after BoNT in different groups of muscles. Hence, if these findings are taken into consideration while injecting BoNT, it could be hypothesized that muscles that have more spindles are dependent on higher sensory input, and when deprived of this input could develop more severe atrophy. Also, the fact that the location of muscle spindles could be either superficial or deep would impact the development of atrophy as superficial spindles would more easily be accessible to BoNT.

## 9. Impact of Muscle Blood Perfusion on Toxin-Induced Atrophy

Calcitonin gene-related peptide (CGRP) is a 37 amino acid peptide and is produced by alternate splicing of RNA transcript of calcitonin gene [79]. Calcitonin gene-related peptide plays a role in muscle vasoregulation and it is a potent vasodilator [80]. Botulinum neurotoxins induce the release of CGRP [80], thereby increasing the average vessel diameter (arterial and venous) which persists for the duration of BoNT-induced muscle paralysis [80,81]. Several studies have shown that restricting blood flow to the muscles decreases the amount of atrophy following chronic unloading of muscles [82]. Another study demonstrated that repetitively restricting the blood flow even with a lower cuff pressure of 50 mmHg decreased the disuse atrophy [83]. The mechanism preventing this development of atrophy is not clear. As the abovementioned studies have shown that restricting blood flow to the muscles decreases disuse atrophy, it could be possible that increased blood flow to muscles following BoNT injections would promote atrophy. Hence, it is likely that blood flow reduction to the injection site, such as regular cooling of the muscle, might reduce atrophy after BoNT injection.

## 10. Why Fat Deposition Occurs after BoNT Injection

Several studies have reported that following multiple and higher doses of BoNT injections, there is evidence of intramuscular lipid accumulation as a pathological response [84,85]. This finding has been seen in a study which showed that at three months after BoNT injection there was presence of fatty infiltration in the muscles which became pronounced at six months [14].

Although the mechanisms are not known, several factors such as activation of satellite cells or alteration of muscle ultrastructure could promote this lipid accumulation.

Botulinum neurotoxins promote adipogenic potential of satellite cells by upregulating adipogenic markers in in vitro experiments [84,85]. Additionally, the adipogenic potential of these SCs is influenced by fiber type and advancing age. Satellite cells isolated from type I muscle fibers have higher adipogenic potential. Also, increasing age can potentiate stem cell differentiation towards an adipogenic fate [85].

Dysferlin, an important muscle membrane protein, is deficient in limb girdle muscular dystrophies which leads to intramuscular lipid accumulation [86,87]. Dysferlin is a SNARE dependent protein and regulates the synaptic acetylcholine receptors (AchR) levels via insertion and retrieval of AchR-containing vesicles at the NMJ. Dysferlin, also regulates the activity of synaptic AchR without changing the number and turnover of AchR [86]. Additionally, treatment with acetylcholinesterase inhibitors such as pyridostigmine can restore synaptic function and aid in muscle strength recovery [88]. Therefore, it appears that dysferlin, a SNARE-dependent protein, has an important role to play in the integrity of the NMJ. Hence, cleavage of SNAREs by BoNT could destabilize dysferlin, thereby muscles accumulate lipid similar to that seen in dysferlinopathies. Since dysferlinopathies are inherited as an autosomal recessive trait, it is possible that carriers of the mutations in this protein would develop more atrophy.

As discussed above, after BoNT injections muscles have the propensity to accumulate lipid which may lead to underestimation of atrophy in these muscles. Therefore, after several BoNT injections it would be prudent to use electromyographyically or ultrasonographically guided injections in order to better localize the bulk of the muscle fiber that has more lipid deposition. 

## 11. Conclusions

In this review, we have highlighted the important yet poorly studied issue of post-BoNT injection atrophy. Despite being known to clinicians, the literature on its presence is scant. Therefore, mechanisms of production of atrophy are also not well studied. Botulinum toxins have been in clinical use for over three decades and have been approved for many neurological and non-neurological indications. It is possible that the study of factors that contribute to atrophy could add valuable information to further optimize the beneficial effects of BoNT injections. As an approach to such factor identification, we have summarized some of them in Table S1 and Figure S1. We have also made suggestions, which the injector might need to take into consideration, as the complex interplay between these factors may determine the development of atrophy.

Our review has certain strengths and weaknesses. This is the first review which has tried to extensively and exhaustingly define the mechanisms behind this common yet ignored side effect of muscle atrophy after botulinum toxin injections. Despite a lack of literature on this topic, we have tried to include whatever sparse data was available based on human and animal studies. However, due to this lack of availability of sufficient information and due to conjecture on our part, this review has the intrinsic weakness of not having a large amount of data to draw upon. Therefore, we suggest that the existing data within pivotal efficacy studies of BoNT should be analyzed in light of the abovementioned factors as well as newer prospective studies designed to explore efficacy of BoNT in various clinical disorders should specifically document muscle atrophy and correlate it to the abovementioned factors. Clinical awareness could result in implementable interventions to not only reduce toxin-induced atrophy but further optimize BoNT therapy.

## Supplementary Materials

The following are available online at http://www.mdpi.com/2072-6651/10/8/313/s1. Figure S1: Factors influencing botulinum toxin induced atrophy, Table S1: Summary of factors influencing BoNT induced atrophy.
