# Supplementary material for: Botulinum Toxin Induced Atrophy: An Uncharted Territory
Source: Toxins (Basel). 2018 Aug 2;10(8):313. doi: 10.3390/toxins10080313 (PMC6115806; doi:10.3390/toxins10080313)
Supplement: Supplementary file 1 [file toxins-10-00313-s001.pdf]

Supplementary Materials: Botulinum Toxin Induced Atrophy: An Uncharted territory

Mehri Salari, Soumya Sharma and Mandar S. Jog

Table S1. Summary of factors influencing BoNT induced atrophy.

Factors that have an impact on BoNT induced muscle atrophy	The way these factors influence atrophy	Outcome	Recommendations
Type of BoNT	Type of SNAREs<br>Cleavage point<br>Different role in nervous system	BoNT/A produces more atrophy than BoNT/B	Change the type of BoNT injected in susceptible individuals
Advancing age	Type IIb fibers<br>Satellite cells	Age decreases type IIb fibers and satellite cells	Change the toxin type, lower dose of BoNT and increase the interval of injections
Gender	Androgen receptors (AR)<br>Estrogen	AR produce a fiber type shift to type I; Estrogen upregulates IGF-1 gene expression	Men may require less amount of BoNT compared to women especially post- menopausal women
Obesity	Fiber type	Decreased amount of type I fibers	Obese individuals may require less amount of toxin
Muscle re-innervation	Fiber type	Re-innervation of type IIb fibers is slower	Muscles such as vastus lateralis and SCM that have higher amount of type IIb fibers require less amount of BoNT
Muscle characteristic	Location<br>Innervation<br>Embryonic origin	a. Proximal muscles re-innervate earlier than distally innervated muscles<br>b. Cranially innervated muscles have higher satellite cells than spinally innervated muscles	Inject less amount of BoNT in distally innervated and spinally innervated muscle
Underlying co-morbidities	Mitochondria<br>Muscle ultrastructure	Mitochondrial cytopathy, alteration in mitochondrial morphology and number, and muscle ultrastructure can influence atrophy	Inject cautiously in patients with overt mitochondrial disease and in diabetics and obese individuals who have mitochondrial dysfunction;<br>Obtain a family history of muscle diseases
Muscle spindles	Number<br>Depth	Muscles having superficial location and higher number of muscle spindles are more susceptible to BoNT effects	Inject less mount of BoNT in vulnerable muscles

Muscle blood perfusion	Vessel diameter	CGRP induced vasodilation	Probably restricting blood flow to injected muscles and using CGRP antagonists could reduce atrophy
Fat deposition	Fiber type dysferlin	Type I fibers have higher adipogenic potential BoNT can produce a condition such as dysferlinopathy	Underestimation of actual amount of muscle atrophy due to fat deposition; and with repetitive injection we need guidance to inject the muscle fibers and not fat; Inject cautiously in patients with family history of myopathies

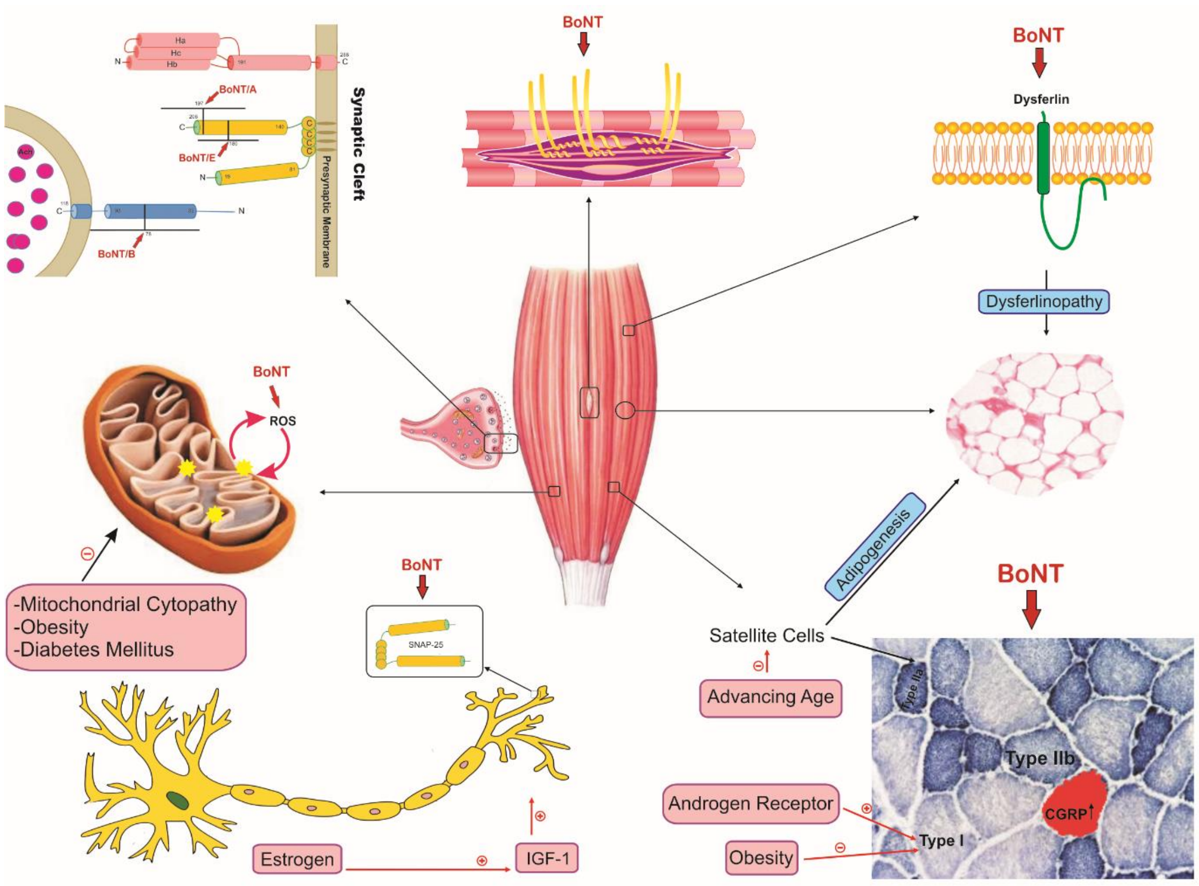

Figure S1. Factors influencing botulinum toxin induced atrophy. Part of the figure was based on figure 3 of article by Pirazzini et al," The Botulinum Neurotoxins: Biology, Pharmacology, and Toxicology".
